# Supplementary material for: Long non‐coding subgenomic flavivirus RNAs have extended 3D structures and are flexible in solution
Source: EMBO Rep. 2019 Sep 10;20(11):e47016. doi: 10.15252/embr.201847016 (PMC6832101; doi:10.15252/embr.201847016)
Supplement: Supplementary file 1 — Appendix [file EMBR-20-e47016-s001.pdf]

APPENDIX

Long non-coding subgenomic flavivirus RNAs have extended 3D structures and are flexible in solution

Yupeng Zhang[1,†] Yikan Zhang[1,†], Zhong-Yu Liu[2, 3, 4,†], Meng-Li Cheng[2, †], Junfeng Ma[1], Yan Wang[1], Cheng-Feng Qin[2*], Xianyang Fang[1, **]

1 Beijing Advanced Innovation Center for Structural Biology, School of Life Sciences, Tsinghua University, Beijing 100084, China

2 State Key Laboratory of Pathogen and Biosecurity, Beijing Institute of Microbiology and Epidemiology, Beijing, 100071, China

3 Guangzhou Eighth People's Hospital, Guangzhou Medical University, Guangzhou, 510060, China

4 School of Medicine (Shenzhen), Sun Yat-sen University, Guangzhou, 510006, China

[*]Corresponding author. Tel: +86 10 66948604; Email: qincf@bmi.ac.cn

[**]Corresponding author. Tel: +86 10 62771071; E-mail: fangxy@tsinghua.edu.cn

[†]These authors contributed equally to this work

Table of Contents

Appendix Table S1. Basic structural parameters for DENV2-, ZIKV-, WNV- sfRNAs at various Mg^{2+} concentrations. Page 3

Appendix Table S2. Basic structural parameters for subdomains of DENV2-, ZIKV-, WNV- sfRNAs at 5mM Mg^{2+} concentrations. Page 4

Appendix Table S3. The R_g and D_{max} values of the conformers in the selected minimal ensemble in comparison with that from ensemble averaged by EOM analysis. Page 5

Appendix Table S4. The primary sequences of full-length and subdomain constructs of DENV2-, ZIKV-, WNV- sfRNAs. Page 6

Appendix Table S5. Primers used for SHAPE analysis. Page 8

Appendix Table S6. SAXS data collection parameters and software employed for data analysis. Page 9

Appendix Table S7. Primers used for plasmid construction and ZIKV detection. Page 10

Appendix Table S1. Basic structural parameters for DENV2-, ZIKV-, WNV- sfRNAs at various Mg^{2+} concentrations.

Sample	$[MgCl_2]$(mM)	[a]R_g (Å)	[b]R_g (Å)	D_{max} (Å)	[c]MW (kD)	[d]MW (kD)	$^{DAMMIN}\chi^2$	NSD^{DAMMIN}
DENV2- sfRNA	1	82.5±1.5	87.7±1.2	313±2	137.21	128.90	n/a	n/a
	2	81.9±1.4	87.7±1.1	310±2		130.18		
	5	79.6±1.1	85.6±1.0	305±2		132.43	0.512±0.159	1.141±0.030
	10	81.7±1.1	85.7±0.9	308±2		145.45	n/a	n/a
ZIKV- sfRNA	1	77.7±2.6	86.2±1.8	316±2	126.86	103.17		
	2	77.1±1.2	83.2±1.3	303±2		103.26		
	5	76.2±1.8	82.0±1.2	295±2		106.25	0.499±0.220	0.975±0.029
	10	77.2±1.3	83.0±1.2	300±2		113.32	n/a	n/a
ZIKV-sfRNA+LNA	5	84.6±2.9	93.2±2.3	346±2	130.90	159.15		
WNV- sfRNA	1	82.9±1.0	89.2±1.1	332±2	161.58	149.26		
	2	81.4±1.0	88.8±1.0	325±2		144.30		
	5	80.4±1.0	86.2±0.8	319±2		150.43	1.065±0.341	1.069±0.030
	10	82.0±1.8	87.9±1.1	322±2		152.08	n/a	n/a

Buffer: 20mM Tris-HCl at pH 7.5, 100mM NaCl; [a] derived from Guinier fitting; [b] derived from GNOM analysis; [c]MW: molecular weight predicted from sequences; [d]MW: molecular weight calculated based on the power law of volume of correlation; n/a: not applicable.

Appendix Table S2. Basic structural parameters for subdomains of DENV2-, ZIKV-, WNV- sfRNAs at 5mM Mg^{2+} concentrations.

Domain	Type	[a]R_g (Å)	[b]R_g (Å)	D_{max} (Å)	[c]MW (kD)	[d]MW (kD)	[e]χ^2	[f]χ^2	[g]χ^2	[h]χ^2	NSD
xrRNA1	DENV2	22.1±0.02	22.5±0.08	80±2	22.17	23.20	0.54			0.084±0.028	0.590±0.019
	ZIKV	24.3±0.2	25.3±0.1	85±2	24.16	22.14	0.82			0.509±0.006	0.659±0.017
	WNV	24.0±0.2	24.9±0.1	84±2	24.20	23.70	0.29			0.521±0.006	0.652±0.022
	MVE	24.3±0.5	26.0±0.2	88±2	26.15	26.28	0.36			0.094±0.001	0.676±0.020
xrRNA2	DENV2	21.9±0.01	22.3±0.06	76±2	22.12	23.54	0.69	n/a	n/a	0.118±0.037	0.661±0.028
	ZIKV	27.3±0.4	28.0±0.2	97±2	22.88	22.71	0.68			0.522±0.006	0.615±0.013
	WNV	22.5±0.2	22.9±0.05	77±2	24.48	24.49	0.74			0.316±0.012	0.610±0.016
	MVE	22.3±0.3	23.0±0.1	82±2	22.61	23.98	0.30			0.109±0.001	0.632±0.022
SL3	WNV	28.09±0.3	29.2±0.07	95±2	24.82	24.68			0.87	0.565±0.010	0.661±0.021
xrRNA1-2	DENV2	35.0±0.2	36.6±0.1	125±2	46.37	43.98		0.44		0.511±0.033	0.830±0.028
	ZIKV	38.5±1.0	39.7±0.3	142±2	49.67	44.65		0.20	n/a	0.151±0.003	0.782±0.025
	WNV	47.4±0.8	48.0±0.3	174±2	75.20	73.57		0.15		0.159±0.024	0.768±0.031
DB12	DENV2	46.1±2.0	48.7±0.4	162±2	56.00	51.40			1.52	0.103±0.004	0.930±0.022
	ZIKV	32.8±0.9	33.1±0.6	112±2	47.90	50.38			0.15	0.097±0.001	0.865±0.038
	WNV	39.2±1.0	39.6±0.4	135±2	58.98	53.34	n/a		0.14	0.158±0.001	0.711±0.026
DB12-PK3[Δ]	ZIKV	43.8±0.9	46.5±0.5	169±2	47.9	50.03			n/a	n/a	0.857±0.023
	WNV	46.4±1.9	49.9±1.5	179±2	58.98	54.35		n/a	n/a	n/a	0.859±0.025
3'SL	DENV2	35.9±0.5	39.4±0.4	142±2	31.47	28.77			0.22	0.191±0.003	0.583±0.021
	ZIKV	39.5±0.9	41.3±0.5	141±2	32.28	27.13			0.18	0.061±0.002	0.654±0.012
	WNV	35.4±0.5	37.9±0.3	132±2	32.22	26.84			0.14	0.136±0.006	0.649±0.010
3'SL-M	WNV	36.8±2.2	40.0±0.4	135±2	32.18	34.98			n/a	0.578±0.004	0.681±0.025

Buffer: 20mM Tris-HCl at pH 7.5, 100mM NaCl, 5mM $MgCl_2$; [a] derived from Guinier fitting; [b] derived from GNOM analysis; [c]MW: molecular weight predicted from sequences; [d]MW: molecular weight calculated based on the power law of volume of correlation; [e]χ^2: ModeRNA models fitting to experimental SAXS data; [f]χ^2: Xplor-NIH rigid-body models fitting to experimental SAXS data; [g]χ^2: Rosetta models fitting to experimental SAXS data; [h]χ^2: DAMMIN bead models fitting to experimental SAXS data; n/a: not applicable.

Appendix Table S3. The R_g and D_{max} values of the conformers in the selected minimal ensemble in comparison with that from ensemble averaged by EOM analysis.

RNA	*Minimal Ensemble*			*Ensemble averaged*	
	Conformers	R_g(Å)	D_{max}(Å)	R_g(Å)	D_{max}(Å)
DENV2-xrRNA12	1	36.41	122.35	33.88	112.94
	2	33.43	111.97		
	3	31.79	104.49		
ZIKV-xrRNA12	1	35.67	123.22	40.13	133.92
	2	44.58	144.62		
WNV-xrRNA12	1	49.22	164.71	47.53	159.98
	2	45.85	155.24		
DENV2-DB12	1	45.43	158.61	46.18	153.97
	2	46.93	149.33		
DENV2-sfRNA	1	89.58	290.29	81.69	261.92
	2	65.05	208.78		
	3	90.45	286.68		
ZIKV-sfRNA	1	99.80	309.49	89.26	290.36
	2	81.31	277.31		
	3	86.66	284.28		
WNV-sfRNA	1	87.91	278.86	85.35	270.43
	2	89.73	289.24		
	3	78.41	243.19		

Appendix Table S4. The primary sequences of full-length and subdomain constructs of DENV2-, ZIKV-, WNV- sfRNAs.

Source	Construct	Primary sequence
DENV2	Full-length (423nt)	^{1}GGAGUCAGGUCGGAUUAAGCCAUAGUAC<br>GGAAAAAACUAUGCUACCUGUGAGCCCC<br>GUCCAAGGACG^{68}UUAAAAGAA^{77}GUCAGG<br>CCAUUACAAAUGCCAUAGCUUGAGUAAA<br>CUGUGCAGCCUGUAGCUCCACCUGAGAA<br>GGUG^{143}UAAAAAAUCU^{153}GGGAGGCCACA<br>AACCAUGGAAGCUGUACGCAUGGCGUAG<br>UGGACUAGCGGUUAGAGGAGACCCCUCC<br>CUUACAAAUCGCAGCAA^{237}CAAU^{241}GGGG<br>GCCCAAGGUGAGAUGAAGCUGUAGUCUC<br>ACUGGAAGGACUAGAGGUUAGAGGAGAC<br>CCCCCCAAAACAAAAAACAGCAU^{324}AUUG<br>A^{329}CGCUGGGAAAGACCAGAGAUCCUGCU<br>GUCUCCUCAGCAUCAUUCCAGGCACAGA<br>ACGCCAGAAAAUGGAAUGGUGCUGUUGA<br>AUCAACAGGUUC^{423}U
	xrRNA1 (68nt)	^{1}G - ^{19}G^{20}C - ^{68}U
	xrRNA2 (69nt)	*GG*^{77}G - ^{94}G^{95}C - ^{143}U
	DB1 (85nt)	^{153}G-^{237}C
	DB2 (84nt)	^{241}G-^{324}A
	3'SL (95nt)	*GG*^{329}C-^{423}U
	xrRNA1-2 (143nt)	^{1}G- ^{74}G^{75}A - ^{143}U
	DB1-2 (172nt)	^{153}G-^{324}A
ZIKV	Full-length (412nt)	^{1}UGUCAGGCCUGCUAGUCAGCCACAGCUU<br>GGGGAAAGCUGUGCAGCCUGUGACCCCC<br>CCAGGAGAAGCUGGG^{72}AAACCAAGCCCA<br>U^{85}AGUCAGGCCGAGAACGCCAUGGCACG<br>GAAGAAGCCAUGCUGCCUGUGAGCCCCU<br>CAGAGGACACUGAG^{153}UCAAAAAACCCCA<br>C^{167}GCGCUUGGAGGCGCAGGAUGGGAAAA<br>GAAGGUGGCGACCUUCCCCACCCUUUAA<br>UCUGGGGCCUGAACUGGAGAUCAGCUGU<br>GGAUCUCCAGAAGAGGGACUAGUGGUUA<br>GAGGAGACCCCCCGGAAAACGCAAAACA<br>GCAUAUU^{312}GACGCUGGGAAAGACCAGAG<br>ACUCCAUGAGUUUCCACCACGCUGGCCGC<br>CAGGCACAGAUCGCCGAAUAGCGGCGGC<br>CGGUGUGGGGAAAUCCAUGGGUC^{412}U
	xrRNA1(72nt)	*GG*^{1}U - ^{72}A
	xrRNA2(69nt)	*G*^{85}A - ^{153}U
	3'SL(98nt)	*G*^{315}G - ^{412}U
	xrRNA1-2(153nt)	^{1}U-^{153}U

	DB12(146nt)	^{167}G - ^{200}GC^{202}G - ^{312}G
	DB12-PK3$^{\Delta}$	^{167}G - ^{200}CG^{202}C - ^{312}G
	LNA	$^{+}$C^{+}G^{+}T^{+}GGGG^{+}TTTTT^{+}T^{+}GA
WNV	Full-length (525nt)	^{1}GGAGUCAGGCCAGAUUAAUGCUGCCACC<br>GGAAGUUGAGUAGACGGUGCUGCCUGCG<br>GCUCAACCCCAGGAGGACUGG^{78}GUGACC<br>AAA^{87}GCUGCGAGGUGAUCCACGUAAGCC<br>CUCAGAACCGUCUCGGAAGGAGGACCCC<br>ACGUGCUUUAGCCUCAAGG^{158}CCCA^{162}GUG<br>UCAGACCACACUCUAGUGUGCCACUCUGC<br>GGAGAGUGCAGUCUGCGAUAGUGCCCCA<br>GGUGGACUGG^{232}GUUAACAAA^{241}GGCAAA<br>ACAUCGCCCCACGCGGCCAUAACCCUGGC<br>UAUGGUGUUAACCAGGGAGAAGGACUA<br>GAGGUUAGAGGAGACCCCGCGUCAAAAA<br>GUGCACGGCCCAACUUGGCUGAAGCUGU<br>AAGCCAAGGGAAGGACUAGAGGUUAGAG<br>GAGACCCCGUGCCAAAAACACCAAAAGA<br>AACAGC^{422}AUAUUGA^{429}CACCUGGGAUAG<br>ACUAGGGGAUCUUCUGCUCUGCACAACC<br>AGCCACACGGCACAGUGCGCCGAUAUAG<br>GUGGCUGGUGGUGCGAGAACACAGGAUC^{525}U
	xrRNA1(78nt)	^{1}G - ^{78}G
	xrRNA2(71nt)	*G*^{162}G - ^{232}G
	SL3(72nt)	*G*^{87}G - ^{158}C
	3'SL(97nt)	*GG*^{429}C - ^{438}UAG^{441}A - ^{525}U
	3'SL-M	*GG*^{429}C - ^{438}GUC^{441}U - ^{525}U
	xrRNA1-2 (232nt)	^{1}G - ^{232}G
	DB12(182nt)	^{241}G - ^{279}GGUG^{283}U - ^{422}A
	DB12-PK3$^{\Delta}$	^{241}G - ^{279}CCAC^{283}A - ^{422}A
MVEV	xrRNA1(80nt)	^{1}GGAGUCAGGCCAGCCGGUUAGGCUGCCA<br>CCGAAGGUUGGUAGACGGUGCUGCCUGC<br>GACCAACCCCAGGAGGACUGGGU^{80}U
	xrRNA2(69nt)	^{1}GGUGUCAGAUCGGCGAAAGUCGCCACUU<br>CGCCGAGGAGUGCAAUCUGUGAGGCCCC<br>AGGAGGACUGGG^{69}U

Appendix Table S5. Primers used for SHAPE analysis.

Primers	Sequences (5′-3′)	5′ modifications	Applications
P-WNV3UTR-R1-FAM	AGATCCTGTGTTCTCGCACCAC	FAM	Primer extension for the SHAPE reactions
P-WNV3UTR-R1-HEX	AGATCCTGTGTTCTCGCACCAC	HEX	Primer extension for the sequencing reactions
P-WNV3UTR-R2-FAM	CCTTTGTTAACCCAGTCCAC	FAM	Primer extension for the SHAPE reactions
P-WNV3UTR-R2-HEX	CCTTTGTTAACCCAGTCCAC	HEX	Primer extension for the sequencing reactions
P-ZIKV3UTR-R1-FAM	CCAGCGTGGTGGAAACTC	FAM	Primer extension for the SHAPE reactions
P-ZIKV3UTR-R1-HEX	CCAGCGTGGTGGAAACTC	HEX	Primer extension for the sequencing reactions
P-ZIKV3UTR-R2-FAM	CACCTTCTTTTCCCATCCT	FAM	Primer extension for the SHAPE reactions
P-ZIKV3UTR-R2-HEX	CACCTTCTTTTCCCATCCT	HEX	Primer extension for the sequencing reactions

Appendix Table S6. SAXS data collection parameters and software employed for data analysis.

Data Collection Parameters		
Facilities and parameters	Settings and values	
Beam line	12ID-B (APS, ANL)	BL19U2 (SSRF)
Wavelength (Å)	0.8857	1.033
Detector	Pilatus 1M (SAXS)	Pilatus 100K (SAXS)
q range ($Å^{-1}$)	0.005-0.89	0.009-0.415
Exposure time (s)	30-60	60
Concentration range (mg/ml)	0.75-3	0.75-3
Temperature (K)	298	298

Software Employed	
Primary Data Processing	Matlab/BioXTAS RAW/PRIMUS
P(r) Function	GNOM
Ab initio Shape Analysis	DAMMIN
SAXS Profile Computation	CRYSOL
Homology modeling	ModeRNA
de novo RNA modeling	Rosetta
Rigid-body Modeling	Xplor-NIH
Flexibility Analysis	EOM/Xplor-NIH
Superposition and averaging	SUPCOMB/DAMAVER
Molecular Visualization	PyMol/Situs/Chimera

Appendix Table S7. Primers used for plasmid construction and ZIKV detection.

Primers	Sequences (5′-3′)	Applications
P-ZIKV/Rep-PK3^-F	CCCCGGAAAAagaAAAACAGCATATTG	Primer for ZIKV-PK3^ or Rep-PK3^ construction
P-ZIKV/Rep-PK3^-R	GGTCTCCTCTAACCACTAG	Primer for ZIKV-PK3^ or Rep-PK3^ construction
P-ZIKV-PK3*-F	cttAATCTGGGGCCTGAACTG	Primer for ZIKV-PK3* construction
P-ZIKV-PK3*-R	cgttAAAGGGTGGGGAAGGTCG	Primer for ZIKV-PK3* construction
P-Rep-PK3*-F	cttAATCTGGGGCCTGAACTG	Primer for Rep-PK3* construction
P-Rep-PK3*-R	cgttGAAGGGTGGGGAAGGTCG	Primer for Rep-PK3* construction
P-Rep-GAA-F	AGTCAGTGGAgcagcaTGCGTTGTGAAGC	Primer for the Rep-GAA construction
P-Rep-GAA-R	GCCATTCGTTTGAGCCTA	Primer for the Rep-GAA construction
P-ZIKV-F	CCGCTGCCCAACACAAG	Primer for detection of viral RNA
P-ZIKV-R	CCACTAACGTTCTTTTGCAGACAT	Primer for detection of viral RNA
P-ZIKV-Probe	6-FAM-AGCCTACCTTGACAAGCA ATCAGACACTCAA-BHQ1	Probe for detection of viral RNA
