# Supplementary material for: Long non‐coding subgenomic flavivirus RNAs have extended 3D structures and are flexible in solution
Source: EMBO Rep. 2019 Sep 10;20(11):e47016. doi: 10.15252/embr.201847016 (PMC6832101; doi:10.15252/embr.201847016)
Supplement: Supplementary file 2 — Expanded View Figures PDF [file EMBR-20-e47016-s002.pdf]

Expanded View Figures

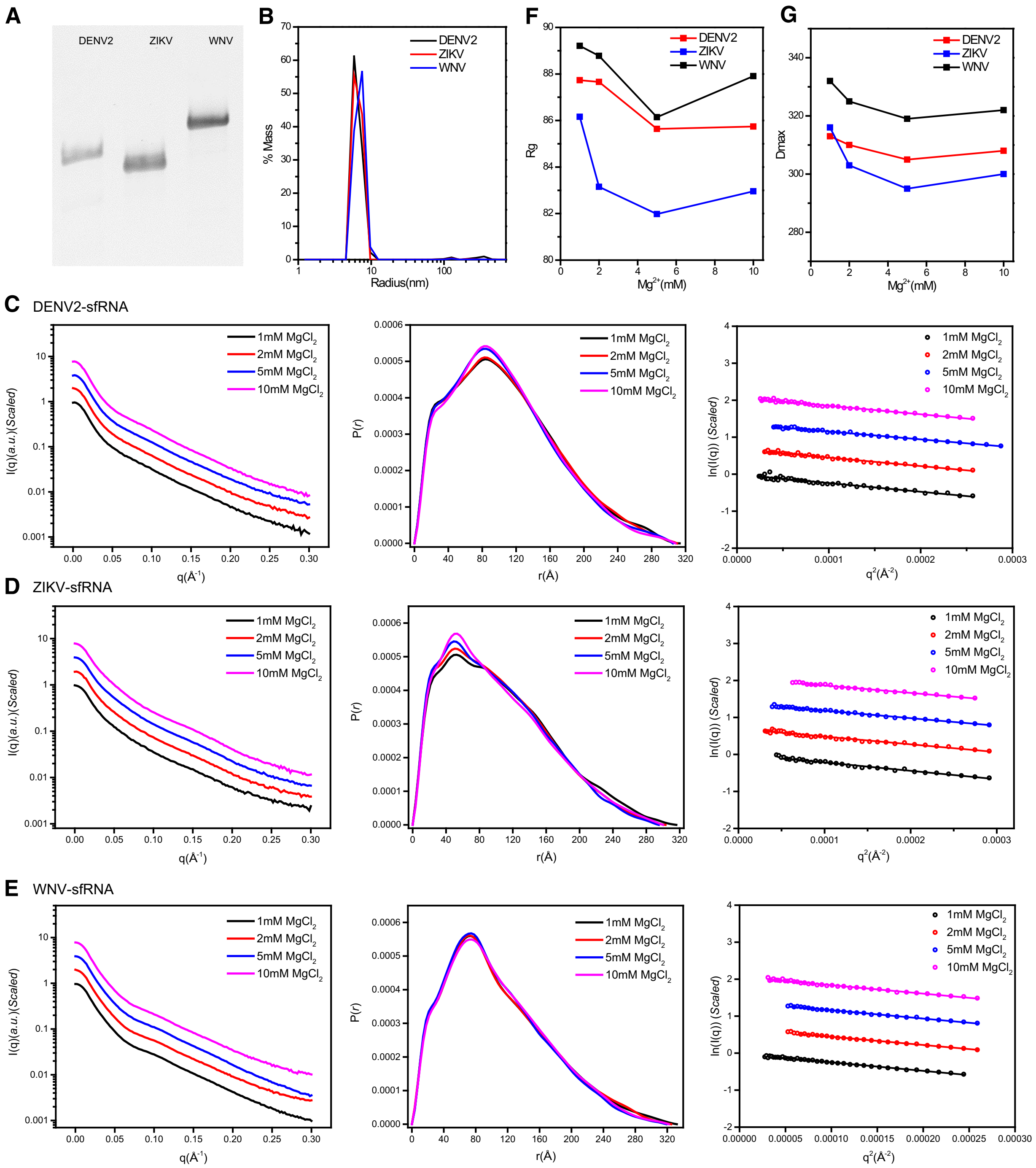

Figure EV1. sfRNA sample preparation and characterization.

A Tris-borate-magnesium (TBM) polyacrylamide gel for sfRNAs of DENV2, ZIKV, and WNV, respectively.

B The distribution of the light scattering by sfRNAs as a function of particle radius in solution.

C–E The scattering curves (left), PDDFs (middle), and guinier plots (right) for sfRNAs of DENV2 (C), ZIKV (D), and WNV (E) at various Mg^{2+} concentrations.

F, G R_g (F) and D_{max} (G) of sfRNAs derived from guinier plots and PDDF calculation were plotted as a function of Mg^{2+} concentration.

© 2019 The Authors

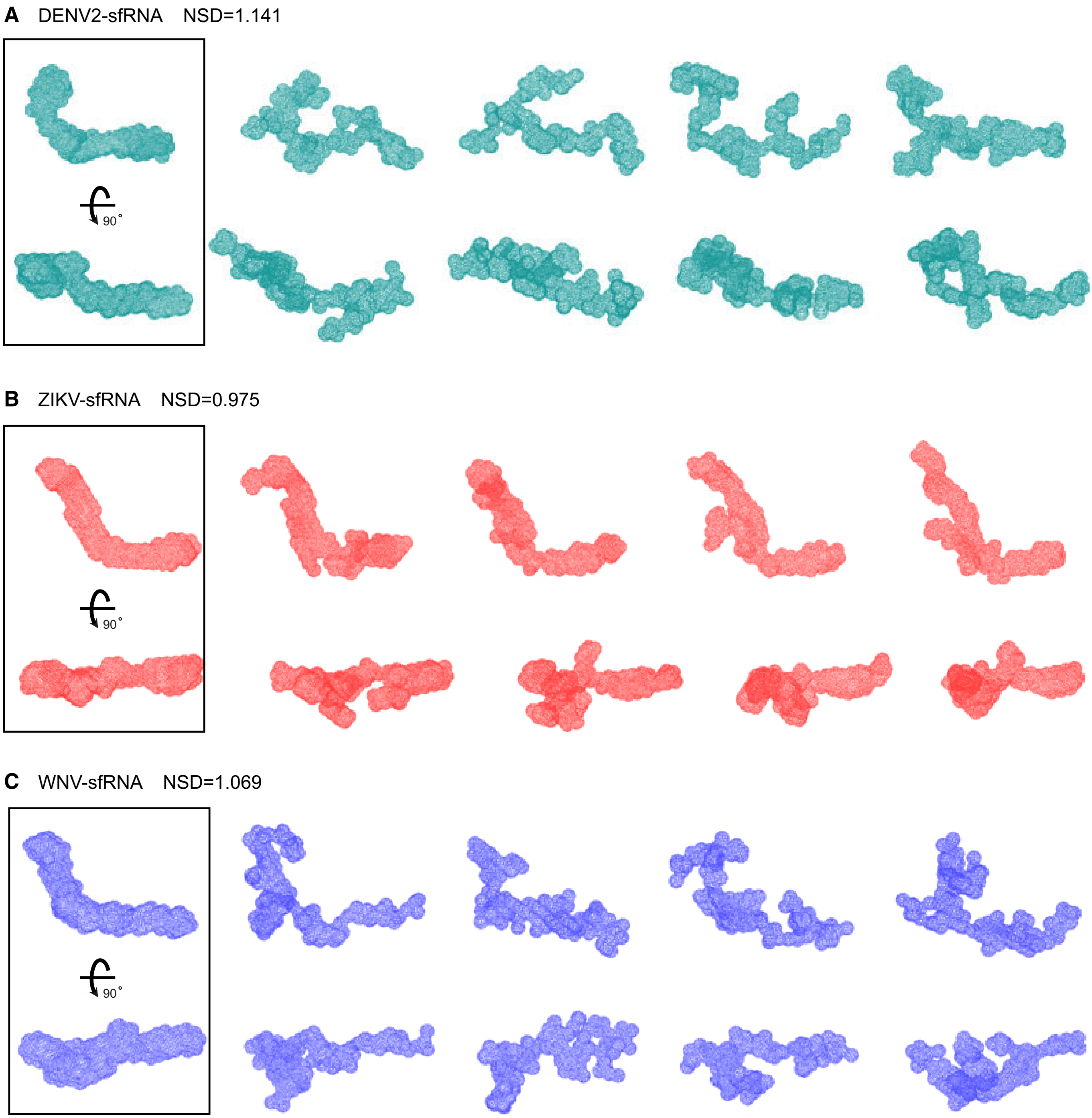

Figure EV2. *Ab initio* modeling of shape envelopes for complete sfRNAs from DENV2 (A), ZIKV (B), and WNV (C).

A–C For each RNA, forty independent *ab initio* shape reconstructions were generated with DAMMIN in slow mode and superimposed with DAMSEL and DAMSUP with NSD values as indicated. Four of 40 individual reconstructions are shown for each RNA in two different orientations, suggesting significant divergence among the shape reconstructions. All 40 superimposed models in each case were averaged and filtered with DAMAVER and DAMFILT to generate the final shape envelopes shown in the boxes on the left.

 © 2019 The Authors

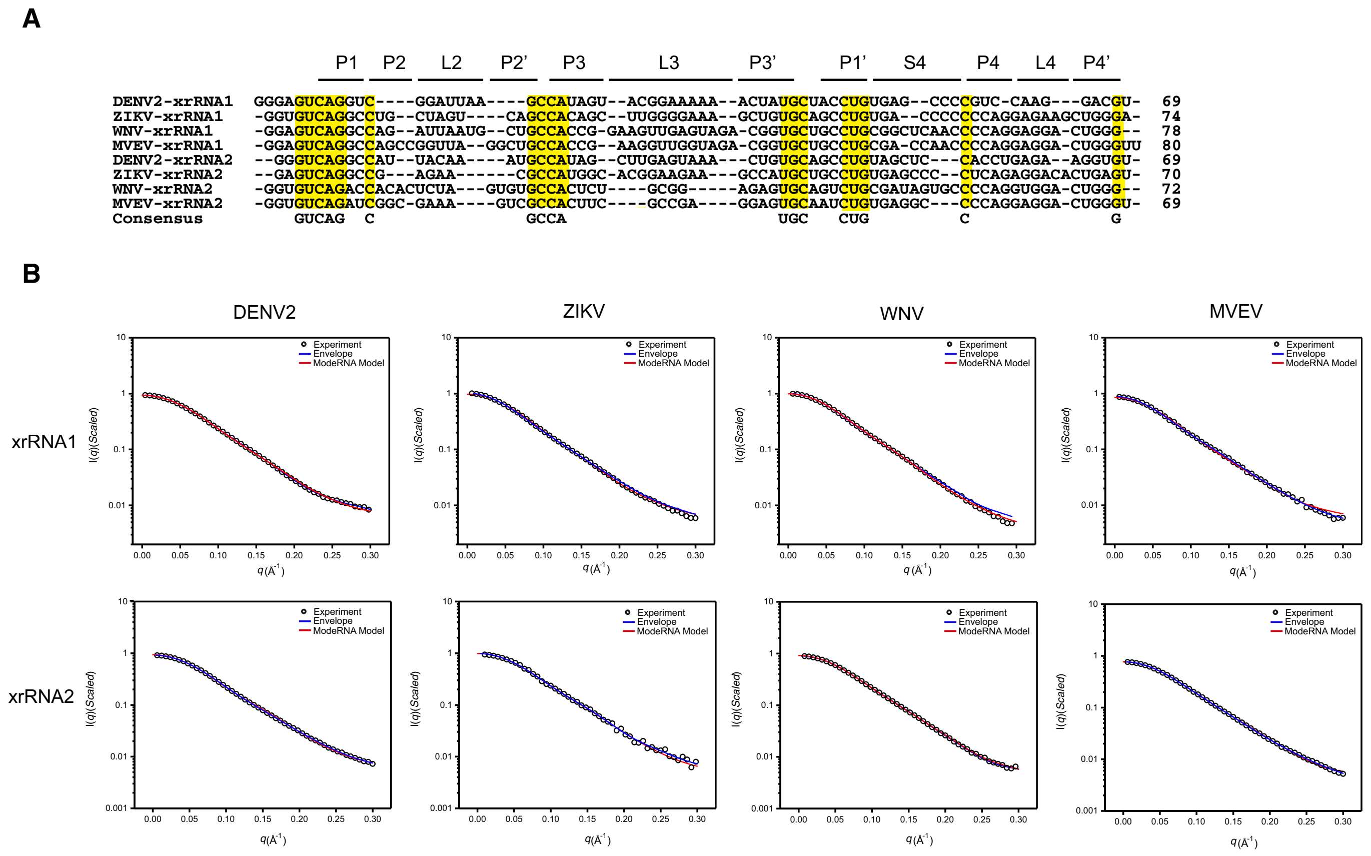

Figure EV3. Primary sequence alignment and fitting of the theoretical to the experimental scattering curves for individual xrRNA1 and xrRNA2.

A Sequence alignment of xrRNA1s and xrRNA2s from DENV2, ZIKV, WNV, and MVEV. The consensus sequences are highlighted in yellow. Sequences in the respective helical or loop regions are indicated at the top.

B Representative fits of the theoretical scattering curves of DAMMIN bead models and ModeRNA atomic models to the experimental SAXS data of xrRNA1s (top row) and xrRNA2s (bottom row) of DENV2, ZIKV, WNV, and MVEV, respectively.

© 2019 The Authors

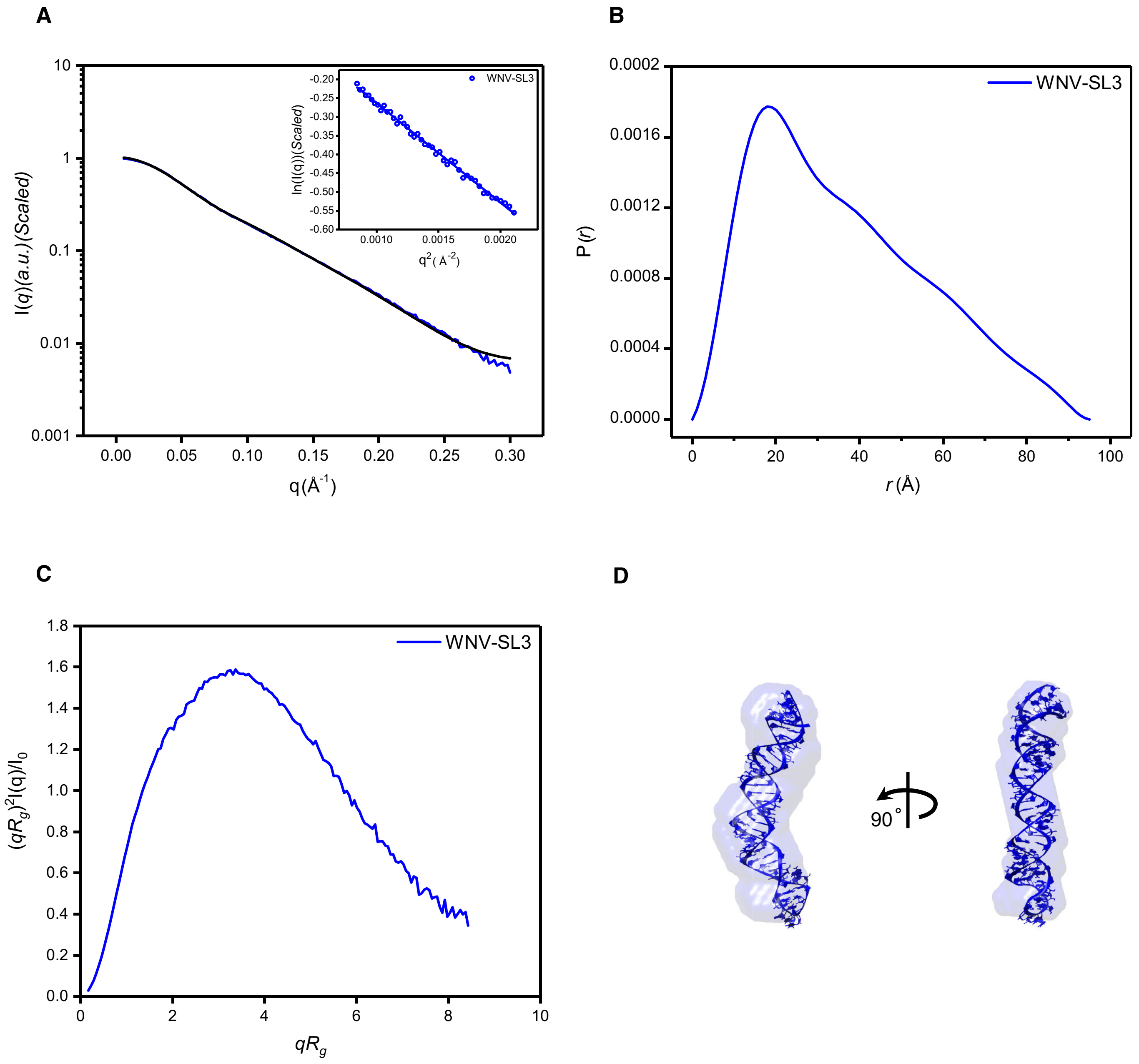

Figure EV4. SAXS characterization and *de novo* structure modeling of SL3 of WNV.

A–C Scattering profile (A), PDDFs (B), and dimensionless Kratky plots (C) of SL3 of WNV. The inset in (A) is the guinier region with fitting line of the scattering profile.

D The *de novo* atomic model by Rosetta was superimposed onto the shape envelope of WNV-SL3. The back-calculated scattering profile of the atomic model (black line) was fitted to the experimental SAXS data of WNV-SL3 (blue line).

© 2019 The Authors

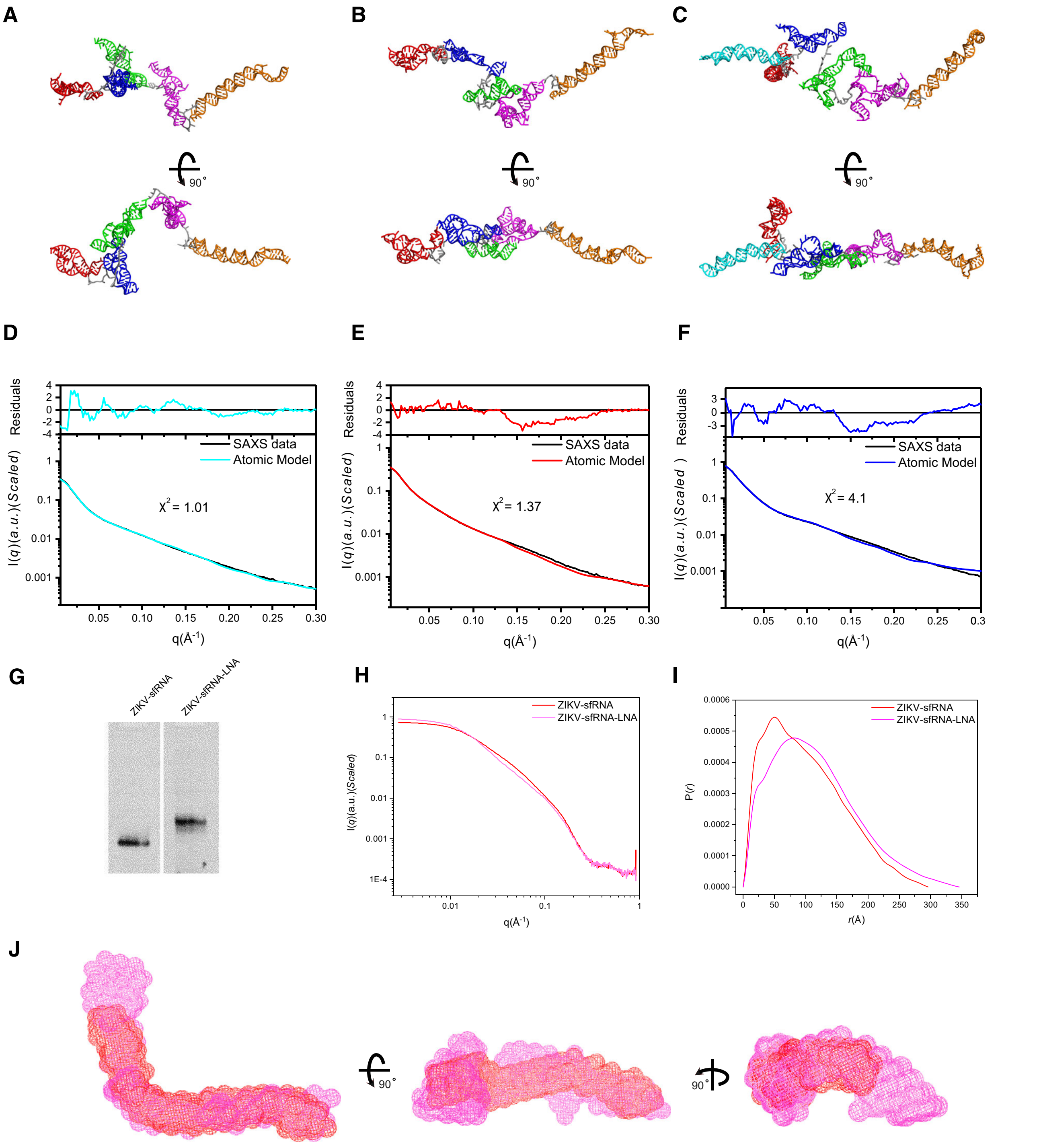

Figure EV5. Atomic models of the complete sfRNAs by rigid-body modeling using Xplor-NIH and LNA binding affect overall structure of ZIKV sfRNA.

A–F Using the available atomic models of the individual subdomains of each sfRNA, all-atom atomic models can be constructed and refined by rigid-body-modeling against SAXS data using Xplor-NIH. The back-calculated scattering curves of the best fit models for DENV2 (A), ZIKV (B), and WNV (C) were fitted to the experimental scattering curves of DENV2 (D), ZIKV (E), and WNV (F), respectively.

G Native PAGE gel for ZIKV sfRNA and ZIKV sfRNA-LNA complex, the latter shows obvious shift retardation relative to ZIKV sfRNA in PAGE gel, indicating that the LNA binding affects overall structure of ZIKV sfRNA.

H, I The differences in scattering profiles (H) and pair distance distribution functions (PDDFs) (I) between ZIKV sfRNA-LNA complex (magenta) and ZIKV sfRNAs (red) indicate that LNA binding affects the overall structure of ZIKV sfRNA significantly. The PDDF profiles in (I) were calculated using GNOM (q_{max} = 0.3).

J Three views of the overlay of the shape envelopes of ZIKV sfRNA-LNA (magenta) onto that of ZIKV sfRNA (red).
